# Supplementary material for: Translocation of Insecticidal Bt Protein in Transgrafted Plants
Source: BioTech (Basel). 2025 Aug 25;14(3):64. doi: 10.3390/biotech14030064 (PMC12452608; doi:10.3390/biotech14030064)
Supplement: Supplementary file 1 [file biotech-14-00064-s001.zip › biotech-3809044-supplementary.pdf]

BioTech

Translocation of Insecticidal Bt Protein in Transgrafted Plants

Arisa Ando [1], Hitomi Ohkubo [1], Hisae Maki [2], Takumi Nishiuchi [3,4], Takumi Ogawa [5], Tomofumi Mochizuki [5], Daisaku Ohta [5], Hiroaki Kodama [1] and Taira Miyahara [1,*]

Supplementary file

Table S1. Non-GM scion leaves used for LC–MS/MS analysis.

Line [a)]	Distance from the graft junction (cm)	
	1st leaf	2nd leaf
WT/Bt44-1	3.3	4.8
WT/Bt44-2	2.4	4.0
WT/Bt44-3	4.5	6.7
WT/Bt44-4	1.0	2.0
WT/Bt44-5	2.0	5.0

The names of the transgrafted plants are consistent with the information of Table 1.

Supplemental methods

Western blot analysis of recombinant proteins from Bt44 plants

For Western blotting, leaves from WT and Bt44 plants were initially homogenized using an extraction buffer containing 200 mM Tris-HCl (pH 8.0), 0.5 mM ethylenediaminetetraacetic acid, and 10 mM 2-mercaptoethanol. The resultant total leaf protein was precipitated with ammonium sulfate. This precipitated protein was subsequently dissolved in 50 mM Tris-HCl (pH 8.0) and desalted using a PD-10 column (Global Life Sciences Technologies Japan, Tokyo) pre-equilibrated with 50 mM Tris-HCl (pH 8.0). Proteins were then separated electrophoretically via SDS-PAGE and transferred onto a nylon membrane. Recombinant Cry1Ab protein was identified by cross-reaction with an anti-Cry1Ab polyclonal antibody (ab51586, Abcam, Cambridge, UK). The membrane was then incubated with a goat HRP-conjugated anti-rabbit IgG secondary antibody (Proteintech Japan, Tokyo, Japan). Visualization of cross-reacted proteins was achieved using Ez-WestBlue (Atto, Tokyo, Japan).

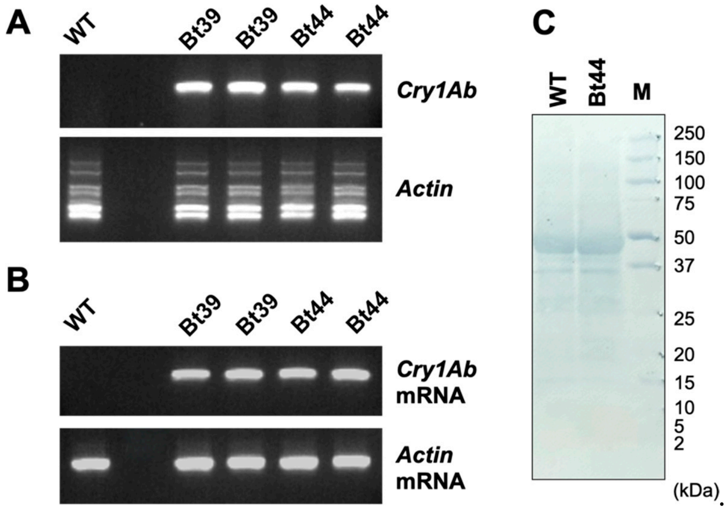

Figure S1. Molecular characterization of *Bt* transformants. (A) Detection of the *Cry1Ab* transgene in Bt39 and Bt44 plant genomic DNA. A 356 bp region of the *Cry1Ab* gene was amplified via PCR. As a control, tobacco Actin genes were amplified; the presence of multiple Actin paralogous genes in the tobacco genome led to the simultaneous amplification of several amplicons with differing lengths. (B) Detection of *Cry1Ab* transcripts via RT-PCR. A 356 bp region corresponding to the *Cry1Ab* transcript was amplified. Fragments of 499 bp amplified from tobacco Actin transcripts served as a loading control for RNA quantity. (C) Western blot analysis for the detection of recombinant Cry1Ab protein. Faint bands visible on the blot were attributed to background-level staining. The recombinant Cry1Ab protein has a predicted molecular mass of approximately 73 kDa. Importantly, no specifically cross-reacting protein band was detected by the anti-Cry1Ab polyclonal antibody.referred to in the content.
